# Development of Poly(HEMA-Am) Polymer Hydrogel Filler for Soft Tissue Reconstruction by Facile Polymerization

**DOI:** 10.3390/polym10070772

**Published:** 2018-07-13

**Authors:** Sujin Kim, Byung Ho Shin, Chungmo Yang, Soohyun Jeong, Jung Hee Shim, Min Hee Park, Young Bin Choy, Chan Yeong Heo, Kangwon Lee

**Affiliations:** 1 Department of Transdisciplinary Studies, Graduate School of Convergence Science and Technology, Seoul National University, Seoul 08826, Korea; sujin.k@snu.ac.kr (S.K.); cmyang88@snu.ac.kr (C.Y.); brightsoohj@snu.ac.kr (S.J.); minheepark@snu.ac.kr (M.H.P.); 2 Department of Biomedical Engineering, College of Medicine, Seoul National University, Seoul 03080, Korea; byungho@snu.ac.kr (B.H.S.); ybchoy@snu.ac.kr (Y.B.C.); 3 Department of Research Administration Team, Seoul National University, Bundang Hospital, Seongnam 13620, Korea; xmylife@empas.com; 4 Interdisciplinary Program for Bioengineering, College of Engineering, Seoul National University, Seoul 08826, Korea; 5 Institute of Medical & Biological Engineering, Medical Research Center, Seoul National University, Seoul 03080, Korea; 6 Department of Plastic and Reconstructive Surgery, College of Medicine, Seoul National University, Seoul 03087, Korea; 7 Department of Plastic and Reconstructive Surgery, Seoul National University Bundang Hospital, Seongnam 13620, Korea; 8 Advanced Institutes of Convergence Technology, Gyeonggi-do 16229, Korea

**Keywords:** Poly(HEMA-Am), hydrogel, soft tissue filler, soft tissue reconstruction

## Abstract

The number of breast reconstruction surgeries has been increasing due to the increase in mastectomies. Surgical implants (the standard polydimethylsiloxane (PDMS) implants) are widely used to reconstruct breast tissues, however, it can cause problems such as adverse immune reactions, fibrosis, rupture, and additional surgery. Hence, polymeric fillers have recently garnered increasing attention as strong alternatives for breast reconstruction materials. Polymeric fillers offer noninvasive methods of reconstruction, thereby reducing the possible adverse effects and simplifying the treatment. In this study, we synthesized a 2-hydroxylethylmethacrylate (HEMA) and acrylamide (Am) copolymer (Poly(HEMA-Am)) by redox polymerization to be used as a biocompatible filler material for breast reconstruction. The synthesized hydrogel swelled in phosphate buffered saline (PBS) shows an average modulus of 50 Pa, which is a characteristic similar to that of the standard dermal acrylamide filler. To investigate the biocompatibility and cytotoxicity of the Poly(HEMA-Am) hydrogel, we evaluated an in vitro cytotoxicity assay on human fibroblasts (hFBs) and human adipose-derived stem cells (hADSCs) with the hydrogel eluate, and confirmed a cell viability of over 80% of the cell viability with the Poly(HEMA-Am) hydrogel. These results suggest our polymeric hydrogel is a promising filler material in soft tissue augmentation including breast reconstruction.

## 1. Introduction

In the field of soft tissue regeneration, the most commonly used medical practice is breast reconstruction using implants after breast tissue resection. Because the number of breast cancer patients has increased, the demand for breast reconstruction operations using implants has also increased [1,2]. However, surgical intervention by silicone implant insertion including polydimethylsiloxane (PDMS) can cause several problems such as implant rupture, infection, hematoma and foreign body reaction. More specifically, a fibrous capsule can cause complications such as capsular contracture or rupture, which could necessitate re-operation and implant removal [2]. Capsular contracture is a significant cause of re-operation because it is a typical side effect of implant surgery. Until now, the mechanism for the development of capsular contracture has not been clearly elucidated, but infection, foreign body reactions, hematoma, and implant content have been reported as causes [3].

Recently, conventional invasive breast reconstruction methods that require incisions such as silicone implant insertion and autologous fat transfer are being replaced by noninvasive breast reconstruction practices. In these procedures, the filler for a local site injection is partially used as a substitute for the implant. Because the demand for tissue augmentation has increased for reconstructive and cosmetic purposes, many different injectable fillers have become available as medical solutions [4].

For cosmetic surgeries, surgeons utilize commercially available soft tissue fillers including hyaluronic acid (HA), collagen, acrylamide (Am) and poly(methyl methacrylate) (PMMA) [5]. Naturally derived, also called temporary, material based fillers have a rapid absorption, and their structures have a relatively low stability due to the high degradation in vivo [6,7,8]. However, the costs associated with these are rather high, even for minimal volumes [9,10]. The HA filler, which is commonly used as a dermal filler, has the problems of fast degradation, high cost, and low stability. It is not feasible to apply a more substantial volume of these fillers in breast reconstruction surgery due to the added cost of the material itself and repeated injections due to the absorption of the material. A collagen filler with PMMA produces a strong immune response because of the PMMA microsphere surface [11], and the Am filler crosslinked with *N*,*N*’-methylenebisacrylamide causes inflammation due to the secondary amine structure [12,13]. Transient adverse reactions to injected bovine collagen have been reported in 1.3% of patients, but recently, temporary injectable fillers containing hyaluronic acid derivatives have been developed for better soft tissue augmentation [14,15]. HA can be modified to form crosslinked polymer molecules that are insoluble and have an extended duration within the tissues. Synthetic fillers have a longer degradation time in vivo compared with naturally derived fillers, and they are composed of permanent or semipermanent substances [16,17]. Injectable liquid silicone, PMMA, and polyimide are examples of such. Nonetheless, they are associated with a high incidence of side effects such as low biocompatibility [18]. Synthetic materials include acrylamide, poly(methylmethacrylate, PMMA), silicone, polycaprolactone (PCL), and poly-l-lactic acid (PLLA) [19]. However, they have the disadvantage of long periods of residual time due to tissue resistance such as a severe inflammatory reaction, granuloma formation, ulceration and migration [20]. One of the other synthetic polymers, acrylamide, is synthesized as a polyacrylamide hydrogel through a network structure using a crosslinker and is used as an injectable hydrogel type filler such as HA and the collagen filler. Nowadays, the most active research is being done in the field of synthetic fillers [21].

In this study, a 2-hydroxylethylmethacrylate (HEMA) and acrylamide (Am) copolymer (Poly(HEMA-Am)) hydrogel were synthesized by redox polymerization as a new synthetic filler. The rapid mixing of the reagents induced the formation of the hydrogel in a few minutes. The modulus and water content of the hydrogel were controlled by manipulating various parameters such as the initial monomer concentration. A water absorption test was used to determine the water content and modulus of the hydrogels, which indicate the suitability of the hydrogel as a soft tissue filler. We evaluated the biocompatibility of the hydrogel eluate by measuring cell viability and cytotoxicity. The results demonstrated that the Poly(HEMA-Am) hydrogel as a synthetic polymer-based filling material is capable of providing a stable structure and biocompatibility that can be used as an injectable filler for breast reconstruction.

## 2. Materials and Methods

### 2.1. Materials

Chemicals were purchased from Aldrich (St. Louis, MO, USA) unless otherwise specified. The solvent was distilled and deionized using a Millipore Milli-Q Ultrapure water purification system (Bedford, MA, USA) at 18.2 M resistance. All reactions were conducted at room temperature (RT). The compound 2-Hydroxyethyl methacrylate (HEMA, 99%) and ethylene glycol dimethacrylate (EGDMA) were passed through a 10-mL syringe packed with a cotton ball, aluminum oxide (Samchun, Korea) and sea sand (Junsei, Japan) to remove the inhibitor hydroquinone monomethyl ether (MEHQ). Aqueous solutions of ammonium persulfate (APS) and tetramethylethylenediamine (TEMED) were used together as redox initiators. Ethylene glycol dimethacrylate (EGDMA) was the crosslinking agent used in the hydrogel polymerization. The dialysis bag (MWCO:5000, Seguin, TX, USA) was purchased from Membrane Filtration (Seguin, TX, USA).

### 2.2. Synthesis of the Poly(HEMA-Am)

The synthesis of the copolymer Poly(HEMA-Am) was performed under water solvent conditions by redox polymerization. Briefly, acrylamide and HEAM were dissolved in deionized (DI) water with EGDMA as a crosslinker, and APS and TEMED were used as redox initiators at RT by stirring them with a magnetic bar under nitrogen gas purging [22]. The specific formulations used are described in Table 1. The hydrogel was gelated and dried under a vacuum for 24 h at 25 °C. After drying the sample to full transparency, the dried hydrogel was left to swell in water for 24 h, and dialysis was performed using a membrane (MWCO:5000, Cellu-Sep T2, USA) to remove the reagents and unreacted monomers.

### 2.3. Fourier Transform Infrared Spectroscopy (FT-IR) of the Poly(HEMA-Am)

Fourier transform infrared spectroscopy (FT-IR) was used to characterize the presence of specific chemical groups in the hydrogels. Poly(HEMA-Am) was obtained in the hydrogel form and analyzed by FT-IR in the attenuated total reflection (ATR) mode. FT-IR spectra were obtained in a wavenumber range from 4000 to 400 cm^−1^ across six scans with a 0.15 resolution (Spectrum GX, Perkin-Elmer, Waltham, MA, USA). KBr pellets of the sample were prepared by mixing 1.5–2.0 mg of dried Poly(HEMA-Am) hydrogel with 200 mg KBr (Sigma, St. Louis, MO, USA) in a vibratory ball mixer for 20 s. [23].

### 2.4. Scanning Electron Microscopy (SEM) of the Poly(HEMA-Am)

Scanning electron microscopy images were obtained with a scanning electron microscopy (mini-SEM) instrument (Mini-SEM SNE 4500 M, SEC, Suwon, Gyeonggi-do, Korea). The SEM images of the Poly(HEMA-Am) by molar ratio were obtained after the Poly(HEMA-Am) hydrogels were quenched in liquid nitrogen at −190 °C and lyophilized. The hydrogel sample was cross-sectioned to obtain an SEM image.

### 2.5. Swelling Properties

The Poly(HEMA-Am) hydrogel was equilibrated in DI water for 24 h, dried in a vacuum, and weighed on a microbalance (Sartorius PT 2100, Sartorius Corporation, Bohemia, NY, USA) to determine the wet mass (*m*_0_). The hydrogel was then dried under a vacuum for 24 h and weighed again, providing the dry mass (*m*). The equilibrium water content of the sample was calculated based on the wet and dry masses of the hydrogel sample according to Equation (1). The average and standard deviation of the four samples were calculated.

The degree of swelling (*W*) was calculated as indicated in Equation (1) [24]:

(1) $$W\left(\%\right)=\frac{m-m_{0}}{m}\times 100$$

where *m* is the weight of the swollen gel in distilled water, and *m*_0_ is the weight of the dry gel under vacuum.

### 2.6. Measurements of the Mechanical Properties

Dynamic shear oscillation measurements at a 10% strain were used to characterize the viscoelastic properties of the Poly(HEMA-Am), standard dermal acrylamide filler (Aquafilling^®^) and human adipose tissue. The rheological measurements at oscillatory shear deformation were carried out with a DHR3 rheometer (TA instruments, New Castle, DE, US) using a 20-mm parallel plate (Peltier plate Steel) with a plate-to-plate distance of 2 mm. Thus, the loaded hydrogel using a 3 mL syringe (BD Science, Franklin Lakes, NJ, USA) and 21 G needle (KOVAX, Seoul, Korea) was about 2.51 mL as a final volume [25]. The mechanical spectra were recorded in the constant strain mode with a deformation of 0.1 maintained over a frequency range of 0.001–1000 Hz (rad/s) at 25 °C. The shear strain dependence of the storage modulus was determined by the oscillatory shear deformation with a shear strain scan ranging from 0.01–100% at a constant frequency (6.3 Hz).

### 2.7. In Vitro Test

***Cell isolation*** All harvested tissues (IRB approval: B-1712-439-304) were sterilized and washed three times in phosphate-buffered saline (PBS). Primary human cells, fibroblasts (FBs) and adipose-derived stem cells (ADSCs) were harvested from abdominal fat tissue. Adipose tissue was collected from abdominal tissue with dermal skin. The upper dermal skin was used for fibroblast isolation. hADSCs were isolated from adipose tissue by washing the tissue sample with PBS containing 1% penicillin/streptomycin. Upon tissue dissection and debris removal, the sample was placed in a sterile tissue culture plate in PBS with 1% penicillin/streptomycin for tissue digestion. Adipose tissue was dissociated using forceps and mixed by pipetting the sample up and down [26].

The adipose tissue was put in a 50 mL sterilized conical tube, and 0.1% collagenase solution was added to the fibroblasts. The cells were incubated in a 0.1% collagenase solution overnight. The sample was incubated for 1 h at 37 °C in a shaking water bath. The digest was centrifuged, thereby separating the floating population of mature adipocytes from the pelleted stromal vascular fraction (SVF). The cell strainer (2.2 μm) was washed with an additional 2 mL of DMEM. After centrifuging three times, the supernatant was removed, and the cells were counted and plated onto a culture plate.

***Cell culture*** hADSCs and hFBs were grown to confluence in high-glucose DMEM with 10% FBS supplemented with penicillin (100 mg/mL) and streptomycin (100 mg/mL). Cells were cultured at the nonpermissive temperature (37 °C) in a humidified atmosphere of 5% CO_2_.

***Cell viability assay*** For the cell viability assay, hADSCs and hFBs were plated onto 24-well plates at a density of 10^4^ cells per well 24 h before the experiment. After dissolving the poly (HEMA-Am) and standard dermal acrylamide fillers in DMEM at a concentration of 0.2 g/mL by ISO 10993-12 [27], they were incubated at 37 °C for 24 h with periodic up-down shaking and filtered by a syringe filter (pore size = 0.22 μm). The 0.2 g/mL hydrogel elute solution was diluted with DMEM at different ratios: 1:1, 1:3, 1:5, 1:10, 1:20, 1:50, and 1:100. The cells were then treated each with the eluate at concentrations for 72 and 120 h. Then, the cells were incubated in a culture medium with the EZ-cytox reagent (DoGen Bio Inc., Seoul, Korea). After incubating for 2 h, each sample was measured at 450 nm using a microplate reader (SpectraMax plus 384; Molecular Devices, Sunnyvale, CA, USA).

***Live/dead assay*** After 72 and 120 h of culturing with the eluate at each concentration, cell viability was assessed with the LIVE/DEAD™ Viability/Cytotoxicity Kit (ThermoFisher, Waltham, MA, USA) including calcein AM to assess the intracellular esterase activity and ethidium homodimer-1 (EthD-1) to assess the plasma membrane integrity [28]. The cells were washed with PBS 3 times, and then, a working solution containing 2 μM calcein AM and 4 μM EthD-1 was added to the washed cells. After a 45 min. incubation at RT in the dark, live cells were observed under an inverted fluorescence microscope (Axio Observer, Carl Zeiss, Oberkochen, Germany).

### 2.8. Statistical Analysis

Statistical significance was determined using the ANOVA test. A *p* < 0.05 indicated statistical significance. The data are presented as the mean and standard deviation for each condition.

## 3. Results and Discussion

### 3.1. Synthesis of the Poly(HEMA-Am)

**Figure 1:**
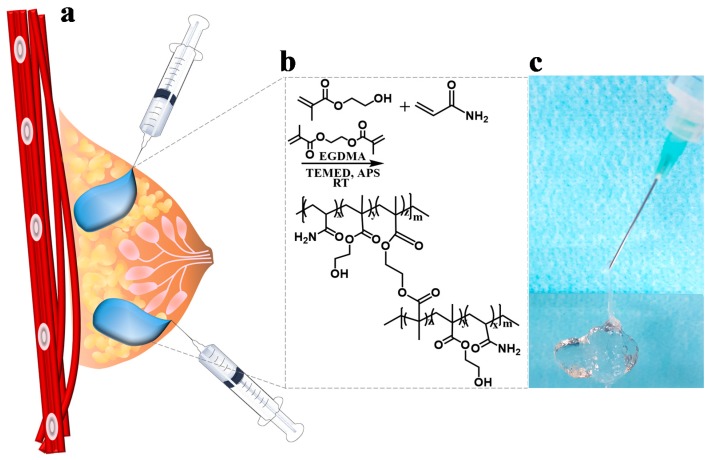
The schematic depicts (**a**) the filling mechanism of soft tissue augmentation by the injectable hydrogel and (**b**) the synthesis method for the Poly(HEMA-Am) hydrogel. (**c**) The photograph shows the potential of the injectable hydrogel at 25 °C.

Figure 1a shows the filling mechanism using the injectable hydrogels as a filler to augment soft tissue including breast reconstruction. To develop synthetic polymer-based hydrogels, we synthesized the Poly(HEMA-Am) hydrogel as an injectable filler by redox polymerization which included Am and HEMA as the monomers and EGDMA as the crosslinker. Redox polymerization, a radical reaction, rapidly yields a hydrogel upon addition of the crosslinker and redox reagents (Figure 1b). By using a lower concentration of monomer and solvent, a more gel-phase characteristic, rather than a brittle property, for the hydrogel can be achieved. In the case of the monomers, the ratio of Am to HEMA determines the physical properties of the hydrogel [29]. In other words, Am has an excess monomer ratio and plays a role in determining the hydrogel properties of the copolymer. These ratios were adjusted to identify the hydrogel with the optimal properties for injectability by syringe (Figure 1c). APS and TEMED were the redox polymerization agents used for the oxidation and reduction, respectively.

The use of inexpensive, commercially and readily available monomers and crosslinking materials enables convenient clinical evaluation and easy mass production. Am is widely used in clinics along with Am-based filler, and HEMA hydrogel is a biocompatible biomaterial used in lenses and bioapplication research on neurons. EGDMA circumvented the existing safety issue by applying a crosslinker with an ester group instead of an amine group because the crosslinker *N*,*N*’-methylenebisacrylamide used in standard dermal acrylamide fillers causes an immune response due to the secondary amine structure [30]. In this study, we were careful to choose conditions that favored an injectable filler.

### 3.2. Characterization of the Poly(HEMA-Am)

#### 3.2.1. FT-IR Spectroscopy

**Figure 2:**
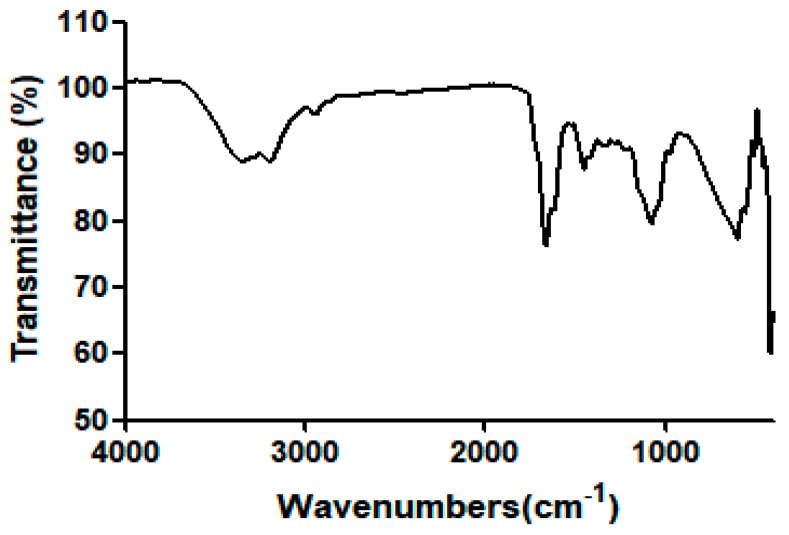
The FT-IR spectra of the Poly(HEMA-Am) hydrogel by ATR mode.

The Poly(HEMA-Am) was characterized by FT-IR spectroscopy. The absorption band at 3347.47 cm^−1^ was assigned to the primary amine groups in the copolymer. The bands at 1662.95 cm^−1^ were the C=O bonds of an amide group and the bending peaks at 1453.39 and 1342 cm^−1^ were the N–H bonds of the amide groups. However, a secondary amine peak (~3600 cm^−1^) was not observed because the Poly(HEMA-Am) does not have a secondary amine structure. This primary amine structure has a higher biocompatibility than that of the standard dermal acrylamide filler which has a secondary amine structure from using the *N*,*N*-1-methylene-bis-acrylamide-based crosslinker. Figure 2 and Table 2 present the Poly(HEMA-Am) hydrogel structure. The structure shows that the Poly(HEMA-Am) has amide bonds but not secondary amine bonds. Additionally, the hydrogel has secondary amide bond crosslinking rather than the primary amine bond crosslinking present in other acrylamide-based fillers. This hydrogel has a nonsecondary amine structure, which is in contrast with another standard dermal acrylamide fillers such as Aquafilling^®^ [4,31]. Thus, the Poly(HEMA-Am) hydrogel has more biocompatible properties than that of other acrylamide-based hydrogels as a filling material because there is no secondary amine structure in the Poly(HEMA-Am).

#### 3.2.2. Scanning Electron Microscope

**Figure 3:**
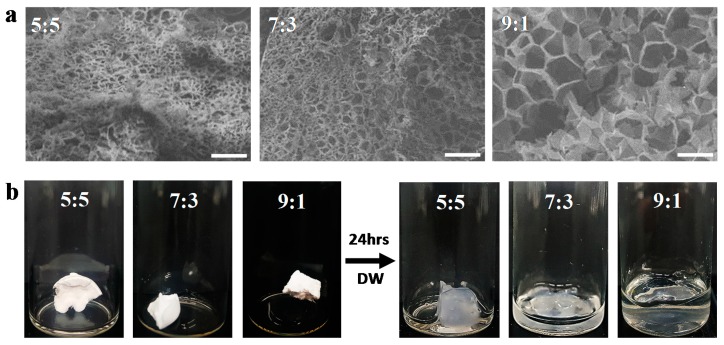
(**a**) The SEM images of the Poly(HEMA-Am) hydrogels prepared with different molar ratios of monomers. The hydrogel was quenched in liquid nitrogen (−196 °C) followed by freeze-drying. The scale bar is 50 μm. (**b**) The photograph shows the swelling behavior of the hydrogel for the different molar ratios before and after soaking in DI water for 24 h. The 5:5, 7:3, and 9:1 indicate the molar ratio of the Am to HEMA monomers, respectively.

The SEM images of the Poly(HEMA-Am) hydrogels were obtained by quenching in liquid nitrogen (–190 °C), followed by freeze-drying the hydrogels. The hydrogels show highly porous structures of the Poly(HEMA-Am) with the different molar ratios of Am to HEMA including the 5:5, 7:3, and 9:1 ratios (Figure 3a). As the Am molar ratio was increased, the pore size increased. The pores are occupied by water molecules, and their sizes affect the injectability and physical properties of the hydrogel. When the concentration of Am is higher than that of HEMA, the degree of gel expansion increases (Figure 3b) and, therefore, the modulus of the hydrogel can be decreased because the porosity size can be controlled by inducing a porous structure in the hydrogel.

### 3.3. Equilibrium Water Content of the Poly(HEMA-Am)

**Figure 4:**
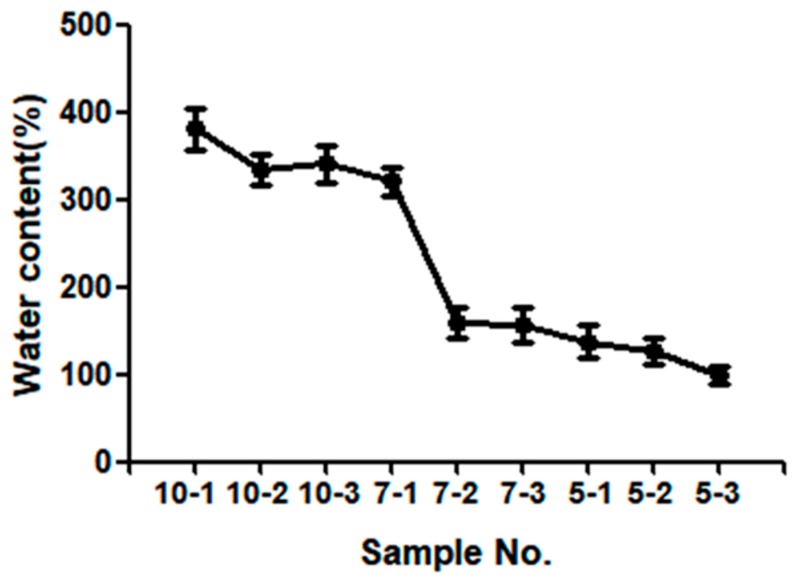
The swelling properties of the Poly(HEMA-Am) by molar ratios of monomers after soaking in DI water for 24 h.

Hydrogel properties, such as swelling and viscoelasticity, are dependent on the HEMA concentration to the total monomer concentration. The ratio of monomer to solvent (Table 1) affected the swelling properties of the Poly(HEMA-Am) hydrogel. In Figure 4, the water content was more than 150 wt %, and samples No. 10-1, 10-2, 10-3 and 7-1 showed a significant increase to 400 wt %. The swelling properties of the hydrogel show that as the concentration of monomers, particularly the concentration of HEMA, increases, the water content decreases. As shown in the SEM image of Figure 3, a gradual increase of the HEMA molar ratio out of the total monomer causes the pore size of the hydrogel to decrease. Especially, we empirically found out that concentration change in between 7-1 and 7-2 is the critical point where the swelling property of the hydrogel dramatically changes. This is also backed up Reference [32] where a critical fluctuation in the gel is caused in terms of local variations in the density of the network. Other than the molar ratio, concentration also affects pore size. In Table 1, though the identical molar ratio of HEMA is present in sample 10-2, 7-2, 5-2, different molar concentrations of the reactant cause different resulting swelling properties. The hydrogel evaluated exhibited considerable water retention and swelling properties. These characteristics decreased the hydrogel gelation time, ultimately leading to the rapid gelation of the Poly(HEMA-Am) hydrogel, which conserved the mechanical properties of the hydrogel even upon contact with a saturated physiological environment [33]. Because the initial hydrogel possessed a water content below its equilibrium value, a swelling process in water is required for the hydrogel to be used as a filler in clinical practice. Because the swelling process expands the filler for tissue repair, it is easy to repair a large volume of tissue with a small amount of hydrogel, and biocompatibility can be ensured. These results show that the Poly(HEMA-Am) hydrogels are promising materials for tissue reconstruction requiring a large volume.

### 3.4. Mechanical Properties of the Poly(HEMA-Am) Hydrogel

**Figure 5:**
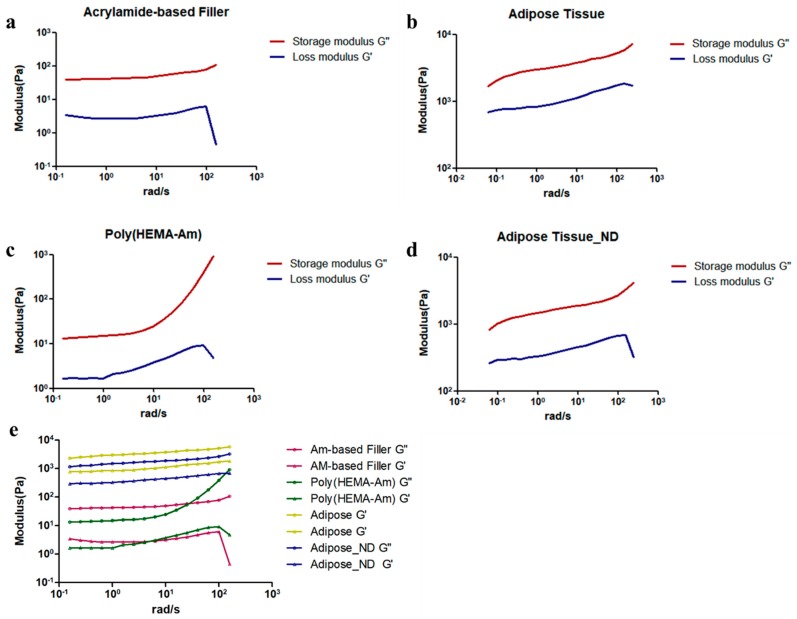
The viscoelastic storage and loss modulus of various samples of the (**a**) acrylamide-based filler, (**b**) adipose tissue with dermis, (**c**) the Poly(HEMA-Am) hydrogel, (**d**) adipose tissue without dermis, and (**e**) comparison of the storage modulus (*G*″) and loss modulus (*G*′). The linear viscoelastic limit of the hydrogel was measured with a frequency sweep of 0.01 Hz.

The mechanical properties of the Poly(HEMA-Am), standard dermal acrylamide filler (Aquafilling^®^) and human adipose tissue with and without dermis were measured by a rheometer. For materials that must retain their structure, the most important factor associated with their development is their mechanical properties. For hydrogel synthesis, controlling and sustaining the mechanical properties required for each designated application are most important because the characteristics of hydrogels change upon contact with water. Thus, we hypothesized that the Poly(HEMA-Am) hydrogel possessed a sufficient modulus to maintain the dissected, hollow part of a surgical location as a tissue reconstruction material [34]. We observed a similar modulus movement by monitoring the evolution of both the storage (*G*′) and loss (*G*″) modulus of the acrylamide-based filler (Figure 5a) and the Poly(HEMA-Am) hydrogel (Figure 5c). The properties of the Poly(HEMA-Am) with a high water content were most appropriate for injectability among the combinations of the various formulas used in the above synthesis. Likewise, the *G* value, which is the sum of the storage modulus and loss modulus, also had a similar value (Pa) and behavior between the Poly(HEMA-Am) hydrogels and acrylamide-based filler (Figure 5e). Figure 5b,d show the modulus of the adipose tissue with and without the dermis for autologous breast reconstruction surgery [35], which was approximately 1 kPa [36] while the adipose tissue with the dermis shows a slightly higher modulus. The viscoelastic properties were determined by monitoring the *G*′ and *G*″ modulus as the strain on the hydrogel increased (Figure 5e), and all the samples showed a high *G*′ value, which is indicative of highly viscoelastic materials. Thus, we identified the potential for the commercial application of the Poly(HEMA-Am) by comparing its mechanical properties with that of a standard dermal acrylamide filler such as Aquafilling^®^.

Figure 5b shows the modulus of unaltered abdominal adipose tissue, and Figure 5d shows the modulus of fat tissue with the dermis removed. Such adipose tissue has a modulus of 1 kPa, which is distinct from that of the Poly(HEMA-Am) and acrylamide-based filler. However, the hydrogel must have an injectable modulus to reach the initially targeted sites; thus, we believe the use of adipose tissue to determine the practicality of the hydrogel is inadequate. Instead, comparison with a commercially available filler would be more logical. From the results, we assert that the Poly(HEMA-Am) hydrogel has a high potential as an injectable filler for soft tissue augmentation by controlling the monomer proportions, which ultimately controls the mechanical properties.

### 3.5. In Vitro Cytotoxicity Assay

#### 3.5.1. Cell Viability Assay

**Figure 6:**
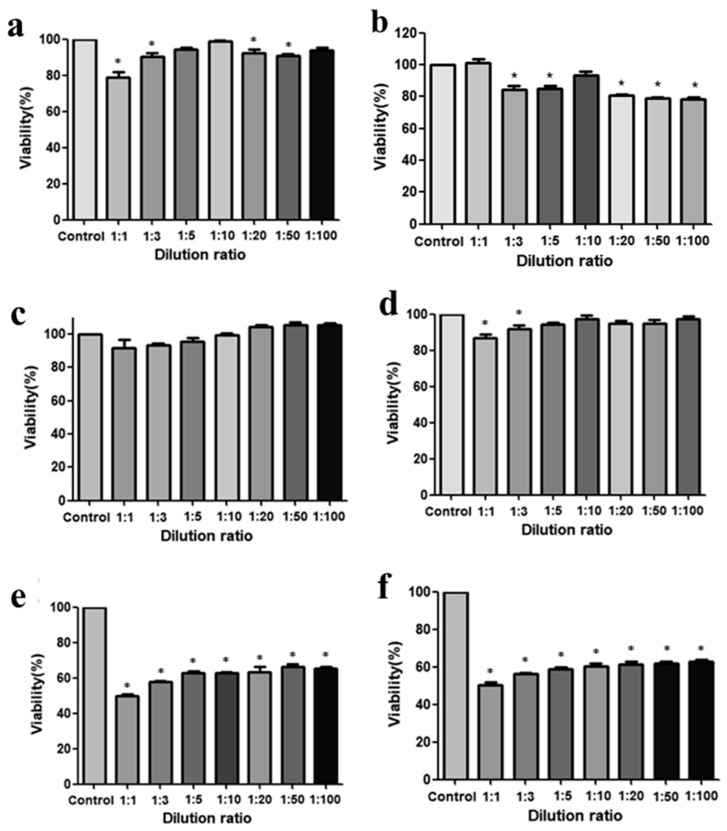
The cell viability assays. The Poly(HEMA-Am) hydrogel and Aquafilling^®^ affect the viability of human fibroblasts and human ADSCs in a concentration-dependent manner. (**a**,**b**) ADSC cytotoxicity following exposure to the Poly(HEMA-Am) hydrogel at 72 and 120 h; (**c**,**d**) fibroblast cytotoxicity following exposure to the Poly(HEMA-Am) at 72 and 120 h; and (**e**,**f**) fibroblast cytotoxicity following exposure to acrylamide-based Filler at 72 and 120 h. * *p* < 0.05 versus the 24 h control. The asterisks (*) represent statistically significant differences compared with the control group.

**Figure 7:**
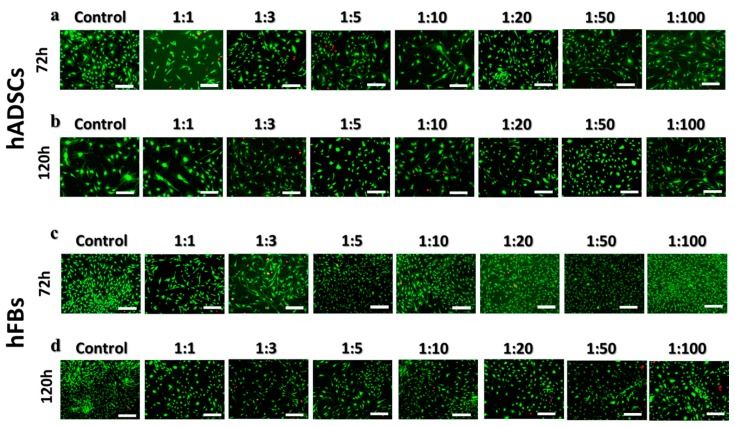
The live/dead fluorescence images of cells treated with the diluted the Poly(HEMA-Am) hydrogel eluate at different ratios. The cytotoxicity of the hADSCs and hFBs at (**a**,**c**) 72 h and (**b**,**d**) 120 h was observed by an inverted fluorescence microscope, respectively. The scale bar is 200 μm.

To evaluate the biocompatibility of the Poly(HEMA-Am) hydrogel, we tested its cytotoxicity by detecting the WST absorbance using the EZ-cytox assay [37]. The hADSCs and hFBs were isolated from abdominal adipose tissue used in a patient’s breast reconstruction, and the cells were cultured and used for the cytotoxicity experiments. Figure 6a,b show the viability of the hADSCs at 72 and 120 h after treatment with the hydrogel eluate diluted with DMEM at different rations: 1:1, 1:3, 1:5, 1:10, 1:20, 1:50, and 1:100, respectively. The cell survival rate was approximately 80%, even with a high concentration of hydrogel eluate at 72 and 120 h. The hFBs showed a higher survival rate of more than 85% to 90% at 72 and 120 h (Figure 6c,d), respectively. Figure 7e,f show the cytotoxicity results of the acrylamide-based filler with cell survival rates of 60–80%, which are lower than those for the Poly(HEMA-Am) hydrogel. From these results, we confirmed that the cell viability was sustained at over 80%, regardless of the hydrogel eluate dilution factor (Figure 6a–d). The results show that the Poly(HEMA-Am) hydrogel is a biocompatible material as a filler that can be used in breast reconstruction compared with the conventional acrylamide filler.

#### 3.5.2. Live and Dead Assay

The cytotoxicity of the Poly(HEMA-Am) hydrogel was evaluated by a live and dead assay of the hADSCs and hFBs using fluorescence microscopy [38]. Figure 7a,b show the fluorescence images of live (green) and dead (red) cells of the hADSCs at each dilution factor after 72 and 120 h of treatment with the hydrogel eluate. Figure 7c,d show the fluorescence images of the hFBs after 72 and 120 h of treatment with the diluted hydrogel eluate. The cell viability was highly conserved, even comparable to that of the control, with little difference between the dilution factors. The notable biocompatibility of the Poly(HEMA-Am) hydrogel was confirmed by the lack of visible dead cells in the figures. We observed a slight decrease in the cell number for both the hADSCs and hFBs by comparing images at 72 and 120 h after the treatment. After 72 h, the decrease in the cell number was attributed to the cell culture being dense, which can inhibit cell proliferation and metabolism. Thus, we determined that the Poly (HEMA-Am) hydrogel is a biocompatible polymer that can be used as a filling material evident by the viability and cytotoxicity results of the hADSCs and hFBs.

## 4. Conclusions

In this study, we successfully synthesized the Poly(HEMA-Am) by redox polymerization as a synthetic polymer-based filling material for breast reconstruction. The chemical structure of the polymer and internal structure of the hydrogel were confirmed by FT-IR and SEM, respectively. The porosity and the mechanical property of the hydrogel were controlled by controlling the molar ration of the monomers. We determined that the Poly(HEMA-Am) hydrogel has great potential as an injectable filler for soft tissue augmentation by comparing its physical and mechanical properties with a standard dermal acrylamide filler. Likewise, we confirmed that the Poly(HEMA-Am) hydrogel is biocompatible based on cell viability and cytotoxicity assays. In conclusion, the results of this study suggest that the Poly(HEMA-Am) hydrogel filler is a promising filling material with a stable structure and good biocompatibility that can be used as a permanent injectable filler for breast reconstruction.

## Figures and Tables

**Table 1:** The formulation of the Poly(HEMA-Am) with different molar ratios of monomers.

DI Water (mL)	Sample No.	HEMA (wt % of monomer)	EGDMA (wt % of monomer)	APS/TEMED (wt % of monomer)	Am (wt % of monomer)
10	10-1	18	3.2	1.7/1.7	80
	10-2	31	2.7	1.4/1.4	71
	10-3	40	2.2	1.2/1.2	59
7	7-1	18	3.2	1.7/1.7	80
	7-2	31	2.7	1.4/1.4	71
	7-3	40	2.2	1.2/1.2	59
5	5-1	18	3.2	1.7/1.7	80
	5-2	31	2.7	1.4/1.4	71
	5-3	40	2.2	1.2/1.2	59

**Table 2:** The IR peaks of the Poly(HEMA-Am) hydrogel.

Type of Bond	IR Bands (cm^−1^)
Primary amine (CONH_2_)	3346.71, 3194.65
Amide C=O stretch peak	1660.68
Amide N–H bonding peak	1450.72
Secondary amine stretch peak	-
